# The moderating effect of alternate Mediterranean diet on the association between sedentary behavior and insomnia in postmenopausal women

**DOI:** 10.3389/fnut.2024.1516334

**Published:** 2025-01-07

**Authors:** Zhumei Sheng, Mincong Zhou

**Affiliations:** Department of Women Healthcare, Hangzhou Women’s Hospital, Hangzhou Maternity and Child Health Care Hospital, Hangzhou, China

**Keywords:** sedentary, Mediterranean diet, insomnia symptoms, moderate, NHANES

## Abstract

**Aim:**

The study aimed to explore the moderating role of the alternate Mediterranean diet (aMED) adherence on the association between sedentary behavior and insomnia symptoms in postmenopausal women.

**Methods:**

Data regarding postmenopausal women were obtained for this cross-sectional study from the National Health and Nutrition Examination Survey (NHANES) 2005–2008. Sedentary behavior and insomnia symptoms were assessed using the questionnaire. aMED adherence was evaluated according to 24-h dietary recalls. Weighted univariate logistic regression models were utilized to screen potential covariates. The relationship between sedentary behavior, aMED adherence, and insomnia symptoms was explored using weighted univariate and multivariate logistic regression models. All results were expressed as odds ratios (ORs) and 95% confidence intervals (CIs).

**Results:**

A total of 1,793 postmenopausal women were included in the final analysis. Of them, 643 (37.56%) reported experiencing insomnia symptoms. Among the postmenopausal women, sedentary time of >8 h was associated with insomnia symptoms (OR = 1.41, 95% CI = 1.01–1.96), prolonged nocturnal awakening (OR = 1.38, 95% CI = 1.06–1.79), and undesired early morning awakening (OR = 1.59, 95% CI = 1.09–2.30). No association was observed between adherence to the aMED and insomnia symptoms (OR = 1.05, 95% CI: 0.77–1.44). Among the postmenopausal women with lower adherence to the aMED, the odds of insomnia symptoms were higher in those with sedentary time ≥8 h (OR = 1.63, 95% CI: 1.02–2.62). Similarly, in the participants with low aMED adherence, sedentary time ≥8 h was also associated with prolonged nocturnal awakening (OR = 1.90, 95% CI = 1.27–2.83) and undesired early morning awakening (OR = 1.85, 95% CI = 1.09–3.16).

**Conclusion:**

Adherence to the aMED modulates the association between sedentary behavior and insomnia symptoms in postmenopausal women. Interventions targeting sedentary behavior and dietary patterns may improve sleep quality and overall health in postmenopausal women.

## Introduction

Insomnia is a prevalent sleep disorder characterized by difficulties initiating or maintaining sleep, affecting up to 50% of primary care patients (1). It is a risk factor for impaired functioning, the development of other medical and mental disorders, and increased healthcare costs (2). The incidence of sleep disorders increases progressively with age, especially among postmenopausal women, with approximately 30 to 80% reporting hot flashes and night sweats, which can lead to the onset or worsening of insomnia (3, 4). Therefore, it is important to accurately identify the factors influencing insomnia and implement appropriate interventions to reduce the disease burden in postmenopausal women.

The etiology and pathophysiology of insomnia involve genetic, environmental, behavioral, and physiological factors that culminate in excessive arousal. Studies have reported a link between health-promoting lifestyle behaviors and improved sleep quality (5–7). Sedentary behavior is associated with an increased risk of insomnia, particularly in postmenopausal women (6, 8). It also negatively correlates with melatonin levels, a key regulator of circadian rhythms and sleep (9). Furthermore, the impact of sedentary behavior on sleep may be linked to depression and cardiovascular disease (CVD) (10, 11).

The Mediterranean diet, a widely recognized healthy eating pattern, has been linked to improved sleep (12). High adherence to the alternate Mediterranean diet (aMED) is associated with reduced insomnia symptoms (13). Following the Mediterranean diet may boost melatonin levels and reduce oxidative stress (14). Antioxidant and anti-inflammatory properties help mitigate nervous system inflammation, oxidative nerve damage, and cerebral ischemia (13). Moreover, the Mediterranean diet has a positive impact on metabolic function and mental health (13, 15). Adherence to the Mediterranean diet has also been reported to moderate the relationship between sedentary behavior and obesity in women (16). Building on these findings, we aimed to explore the relationship between sedentary behavior, aMED adherence, and insomnia symptoms in postmenopausal women.

## Methods

### Study design and participants

**Figure 1:**
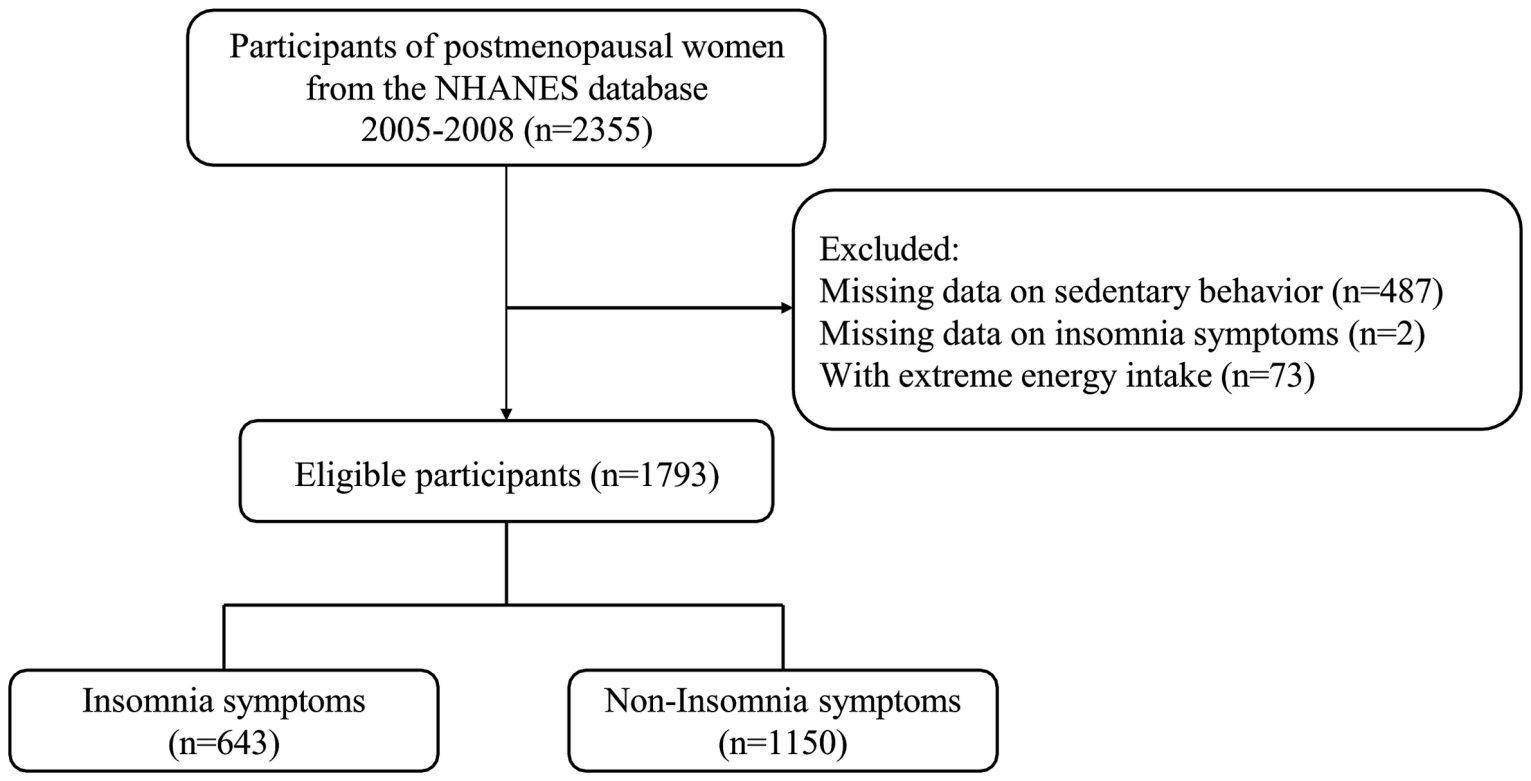
Flowchart of the study’s inclusion and exclusion criteria for postmenopausal women.

Data regarding postmenopausal women for this cross-sectional study were obtained from the 2005–2008 National Health and Nutrition Examination Survey (NHANES), a nationwide survey conducted by the National Center for Health Statistics. Participants underwent health and nutritional assessments, including anthropometric measurements, laboratory tests, and questionnaires. The study was approved by the Research Ethics Committee of the National Public Health Institute. The study protocol did not require approval from the Institutional Review Board of Hangzhou Women’s Hospital. Postmenopausal women with aMED assessment and insomnia symptoms were included. Participants with extreme energy intake (<500 kcal or > 5,000 kcal) and missing key covariates were excluded. Specifically, 487 participants were excluded due to missing data on sedentary behavior, 73 for extreme energy intake, and two for missing insomnia data. Ultimately, 1,793 postmenopausal women were included in the final analysis. The selection process is shown in Figure 1.

### Sedentary behavior

In the NHANES questionnaire, sedentary behavior included sitting at work, at home, during transportation, and while socializing, such as desk work, commuting, reading, playing card games, watching TV, or using a computer, excluding sleep time (17). Sitting for more than 8 h/day was defined as sedentary behavior (18).

### aMED assessment

Adherence to the Mediterranean diet was evaluated using the aMED. Dietary intake data were collected through 24 h dietary recalls. The first recall was conducted during a face-to-face assessment at a mobile examination center (day one), while the second was conducted over the telephone (day 2) within 10 days of the initial interview. To calculate the aMED score, the participants’ intake of vegetables (excluding potatoes), legumes, fruits, nuts, whole grains, red/processed meat, fish, alcohol, and olive oil was assessed (19). The participants who scored above the median intake for fish, whole grains, legumes, nuts, fruits, vegetables, and olive oil received one point. For red/processed meat and alcohol, the individuals were awarded one point if their consumption was below the median or if they had moderate alcohol intake. Those who did not meet these criteria received zero points (20). Higher aMED scores indicated better adherence to the Mediterranean diet.

### Insomnia symptoms

Insomnia symptoms were diagnosed using the NHANES sleep disorders questionnaires, which included three questions regarding the frequency of trouble falling asleep (SLQ080), prolonged nocturnal awakening (SLQ090), and undesired early morning awakening (SLQ100) in the previous month. These symptoms are included in the definition of insomnia in the Diagnostic and Statistical Manual of Mental Disorders, 5th edition (DSM-V) (21). Insomnia is diagnosed when an individual reports experiencing at least one of these symptoms five or more times within the past month (22, 23).

### Covariates

Potential covariates included age, ethnicity, education level, poverty-to-income ratio (PIR), occupation, body mass index (BMI), C-reactive protein, vitamin D, use of female hormones, anxiolytics, sedatives, and hypnotics, depression, history of CVD, cancer or malignancy, smoking status, drinking status, hypertension, diabetes, dyslipidemia, physical activity, energy intake, alcohol consumption, and caffeine intake. During the NHANES household interview, data on age, gender, ethnicity, education level, occupation, use of female hormones, anxiolytics, sedatives, and hypnotics, smoking status, drinking status, energy intake, alcohol consumption, and caffeine intake were collected using questionnaires. The PIR was the ratio of monthly family income to the poverty threshold specific to family size, as released by the Health and Human Services poverty guidelines. BMI was calculated as weight divided by height squared (kg/m^2^). Laboratory tests included C-reactive protein and vitamin D. Vitamin D ≥75 nmol/L was defined as adequate, while <75 nmol/L was considered insufficient. Depression was determined based on a 9-item Patient Health Questionnaire (PHQ-9) score of ≥10 (24). Cancer diagnoses were based on the following questions (25): (1) Have you ever been told by a doctor or other health professional that you had cancer or a malignancy of any kind? (2) What kind of cancer was it, and when was it diagnosed? Hypertension was defined as self-reported hypertension, a systolic bold pressure ≥140 mmHg, a diastolic blood pressure ≥90 mmHg, or the use of antihypertensive drugs (26). Diabetes was defined as self-reported diabetes, a fasting plasma glucose level of 126 mg/dL or more, or a hemoglobin A1c level of 6.5% or more (27). Dyslipidemia was defined as having high triglyceride levels or hypercholesterolemia (28).

### Statistical analysis

Sampling weights (WTMEC2YR, SDMVPSU, and SDMVSTRA) were used in all analyses to generalize results to the U.S. civilian non-institutionalized resident population. The data were presented as count (%) for categorical variables and mean ± standard error (SE) for continuous variables. Comparisons between the two groups were conducted utilizing chi-square tests and *t*-tests. Potential covariates were selected using weighted univariate logistic regression models. The associations between sedentary behavior, adherence to the aMED, and insomnia symptoms were explored using weighted univariate and multivariate logistic regression models. The results were expressed as odds ratios (ORs) with 95% confidence intervals (CIs). All analyses were conducted using SAS 9.4 (Institute Inc., Cary, NC, United States), and *p* < 0.05 was considered statistically significant.

## Results

### Characteristics of the postmenopausal women

Data from 2,355 postmenopausal women, collected between 2005 and 2008, were analyzed. The mean age was 61.95 (0.42) years, with 960 (78.53%) women identified as non-Hispanic White. A total of 327 (18.24%) women reported sedentary time of ≥8 h. Insomnia symptoms were present in 643 (37.56%) women, and 736 (39.38%) exhibited high adherence to the aMED. Statistical differences were found between the two groups in occupation, BMI, physical activity, prolonged nocturnal awakening, and undesired early morning awakening. Table 1 presents the characteristics of the postmenopausal women.

**Table 1:** Characteristics of the postmenopausal women.

Variables	Total (*n* = 1,793)	Sedentary time		*p*
		<8 h (*n* = 1,466)	≥8 h (*n* = 327)	
Age, years, mean (SE)	61.95 (0.42)	61.94 (0.51)	61.95 (0.51)	0.994^a^
Ethnicity, *n* (%)				0.217^b^
Mexican American	254 (4.21)	223 (4.45)	31 (3.18)	
Other Hispanic	154 (3.01)	131 (3.19)	23 (2.24)	
Non-Hispanic White	960 (78.53)	776 (78.91)	184 (76.87)	
Non-Hispanic Black	371 (10.00)	296 (9.69)	75 (11.33)	
Other ethnicity—including multi-racial	54 (4.25)	40 (3.76)	14 (6.38)	
Education level, *n* (%)				0.238^b^
Less than 9th grade	259 (6.95)	227 (7.29)	32 (5.47)	
9–11th grade (includes 12th grade with no diploma)	277 (11.67)	229 (11.76)	48 (11.31)	
High school grad/GED or equivalent	481 (28.40)	393 (29.06)	88 (25.59)	
Some college or AA degree	452 (28.50)	366 (28.54)	86 (28.34)	
College graduate or above	324 (24.47)	251 (23.35)	73 (29.30)	
PIR, mean (SE)	3.13 (0.08)	3.09 (0.08)	3.29 (0.14)	0.074^a^
Occupation, *n* (%)				0.002^b^
A regular daytime schedule	502 (35.45)	380 (33.00)	122 (45.95)	
A regular evening/night shift	44 (2.22)	41 (2.62)	3 (0.51)	
A rotating shift	42 (3.01)	37 (3.13)	5 (2.52)	
Another schedule	63 (4.46)	46 (4.41)	17 (4.68)	
Not working at a job	1,142 (54.86)	962 (56.84)	180 (46.35)	
Body mass index (kg/m^2^), mean (SE)	29.04 (0.23)	28.67 (0.23)	30.63 (0.83)	0.035^a^
C-reactive protein (mg/dL), mean (SE)	0.49 (0.03)	0.47 (0.03)	0.56 (0.07)	0.245^a^
Vitamin D (D2 + D3) (nmol/L), *n* (%)				0.796^b^
Insufficient	1,192 (59.78)	969 (59.55)	223 (60.76)	
Adequate	601 (40.22)	497 (40.45)	104 (39.24)	
Female hormones use, *n* (%)				0.280^b^
No	1,640 (87.99)	1,337 (87.33)	303 (90.82)	
Yes	153 (12.01)	129 (12.67)	24 (9.18)	
Anxiolytics, sedatives, and hypnotics, *n* (%)				0.207^b^
No	1,595 (88.38)	1,315 (88.81)	280 (86.56)	
Yes	198 (11.62)	151 (11.19)	47 (13.44)	
Depression, *n* (%)				0.060^b^
No	1,370 (73.91)	1,137 (74.95)	233 (69.42)	
Yes	423 (26.09)	329 (25.05)	94 (30.58)	
History of CVD, *n* (%)				0.234^b^
No	1,386 (79.95)	1,148 (80.75)	238 (76.53)	
Yes	407 (20.05)	318 (19.25)	89 (23.47)	
Cancer or malignancy, *n* (%)				0.339^b^
Yes	280 (16.91)	231 (17.28)	49 (15.30)	
No	1,513 (83.09)	1,235 (82.72)	278 (84.70)	
Smoking status, *n* (%)				0.348^b^
Yes	277 (15.73)	229 (16.41)	48 (12.82)	
No	1,060 (55.94)	872 (55.49)	188 (57.89)	
Quit smoking	456 (28.33)	365 (28.10)	91 (29.29)	
Drinking status, *n* (%)				0.691^b^
Yes	960 (59.99)	786 (60.27)	174 (58.75)	
No	833 (40.01)	680 (39.73)	153 (41.25)	
Hypertension, *n* (%)				0.657^b^
No	142 (9.27)	119 (9.50)	23 (8.28)	
Yes	1,651 (90.74)	1,347 (90.51)	304 (91.72)	
Diabetes, *n* (%)				0.069^b^
No	1,304 (79.56)	1,074 (80.59)	230 (75.16)	
Yes	489 (20.44)	392 (19.41)	97 (24.84)	
Dyslipidemia, *n* (%)				0.357^b^
No	290 (16.07)	230 (15.64)	60 (17.93)	
Yes	1,503 (83.93)	1,236 (84.36)	267 (82.08)	
Physical activity, *n* (%)				<0.001^b^
<750 MET^b^ min/week	1,088 (55.33)	841 (51.63)	247 (71.21)	
≥750 MET^b^ min/week	705 (44.67)	625 (48.37)	80 (28.80)	
Energy (kcal), mean (SE)	1671.75 (19.28)	1671.03 (20.63)	1674.86 (56.32)	0.951^a^
Alcohol intake (gm), mean (SE)	0.34 (0.03)	0.34 (0.03)	0.37 (0.05)	0.420^a^
Caffeine (mg), mean (SE)	181.84 (8.80)	185.37 (9.07)	166.68 (20.00)	0.369^a^
Difficulty falling asleep, *n* (%)				0.753^b^
No	1,401 (76.81)	1,149 (77.09)	252 (75.62)	
Yes	392 (23.19)	317 (22.92)	75 (24.38)	
Prolonged nocturnal awakening, *n* (%)				0.004^b^
No	1,341 (73.81)	1,108 (75.12)	233 (68.19)	
Yes	452 (26.19)	358 (24.88)	94 (31.81)	
Undesired early morning awakening, *n* (%)				0.015^b^
No	1,401 (79.48)	1,159 (80.86)	242 (73.59)	
Yes	392 (20.52)	307 (19.14)	85 (26.41)	
Insomnia symptoms, *n* (%)				0.072^b^
No	1,150 (62.44)	948 (63.54)	202 (57.73)	
Yes	643 (37.56)	518 (36.46)	125 (42.27)	
aMED, *n* (%)				0.491^b^
Low level	1,057 (60.62)	859 (61.16)	198 (58.31)	
High level	736 (39.38)	607 (38.84)	129 (41.69)	

SE, standard error; PIR, poverty income ratio; CVD, cardiovascular disease; aMED, the alternate Mediterranean diet.

a *t-test.*

b Chi-square test.

### Associations between sedentary behavior, adherence to the aMED, and insomnia symptoms in the postmenopausal women

After adjusting for age, ethnicity, occupation, anxiolytics, sedatives, hypnotics, depression, and smoking status, sedentary time of ≥8 h was associated with increased odds of insomnia symptoms (OR = 1.41, 95% CI: 1.01–1.96). In addition, sedentary time of ≥8 h was also related to a higher risk of prolonged nocturnal awakening (OR = 1.38, 95% CI: 1.06–1.79) and undesired early morning awakening (OR = 1.59, 95% CI: 1.09–2.30) in the adjusted model. No significant association was observed between adherence to aMED alone and insomnia symptoms (OR = 1.05, 95% CI: 0.77–1.44). Table 2 shows the relationships between sedentary behavior, adherence to the aMED, and insomnia symptoms in the postmenopausal women.

**Table 2:** Associations between sedentary behavior, adherence to the aMED, and insomnia symptoms in the postmenopausal women.

Events	Variables	Model 1		Model 2	
		OR (95% CI)	*p*	OR (95% CI)	*p*
Insomnia symptoms	Sedentary time				
	<8 h	Ref.		Ref.	
	≥8 h	1.28 (0.97–1.68)	0.080	1.41 (1.01–1.96)	0.042
	aMED				
	High level	Ref.		Ref.	
	Low level	1.22 (0.91–1.63)	0.173	1.05 (0.77–1.44)	0.747
Difficulty falling asleep	Sedentary time				
	<8 h	Ref.		Ref.	
	≥8 h	1.08 (0.64–1.83)	0.755	1.23 (0.72–2.09)	0.438
	aMED				
	High level	Ref.		Ref.	
	Low level	1.56 (1.13–2.14)	0.008	1.28 (0.88–1.86)	0.192
Prolonged nocturnal awakening	Sedentary time				
	<8 h	Ref.		Ref.	
	≥8 h	1.41 (1.10–1.80)	0.007	1.38 (1.06–1.79)	0.017
	aMED				
	High level	Ref.		Ref.	
	Low level	1.25 (0.90–1.74)	0.176	1.15 (0.83–1.59)	0.402
Undesired early morning awakening	Sedentary time				
	<8 h	Ref.		Ref.	
	≥8 h	1.52 (1.08–2.13)	0.018	1.59 (1.09–2.30)	0.017
	aMED				
	High level	Ref.		Ref.	
	Low level	1.22 (0.81–1.83)	0.331	1.09 (0.73–1.64)	0.655

Ref., reference; OR, odds ratio; CI, confidence interval; aMED, the alternate Mediterranean diet. Model 1 was the crude model. Model 2 was the adjusted model. Insomnia symptoms, adjusted for age, ethnicity, occupation, anxiolytics, sedatives, hypnotics, depression, and smoking status. Difficulty falling asleep, adjusted for age, ethnicity, PIR, occupation, anxiolytics, sedatives, hypnotics, depression, and smoking status. Prolonged nocturnal awakening, adjusted for age, anxiolytics, sedatives, hypnotics, depression, and smoking status. Undesired early morning awakening, adjusted for ethnicity, PIR, occupation, vitamin D, anxiolytics, sedatives, hypnotics, depression, history of CVD, cancer, smoking status, and diabetes.

### Adherence to the aMED affects the association between sedentary behavior and insomnia symptoms

**Figure 2:**
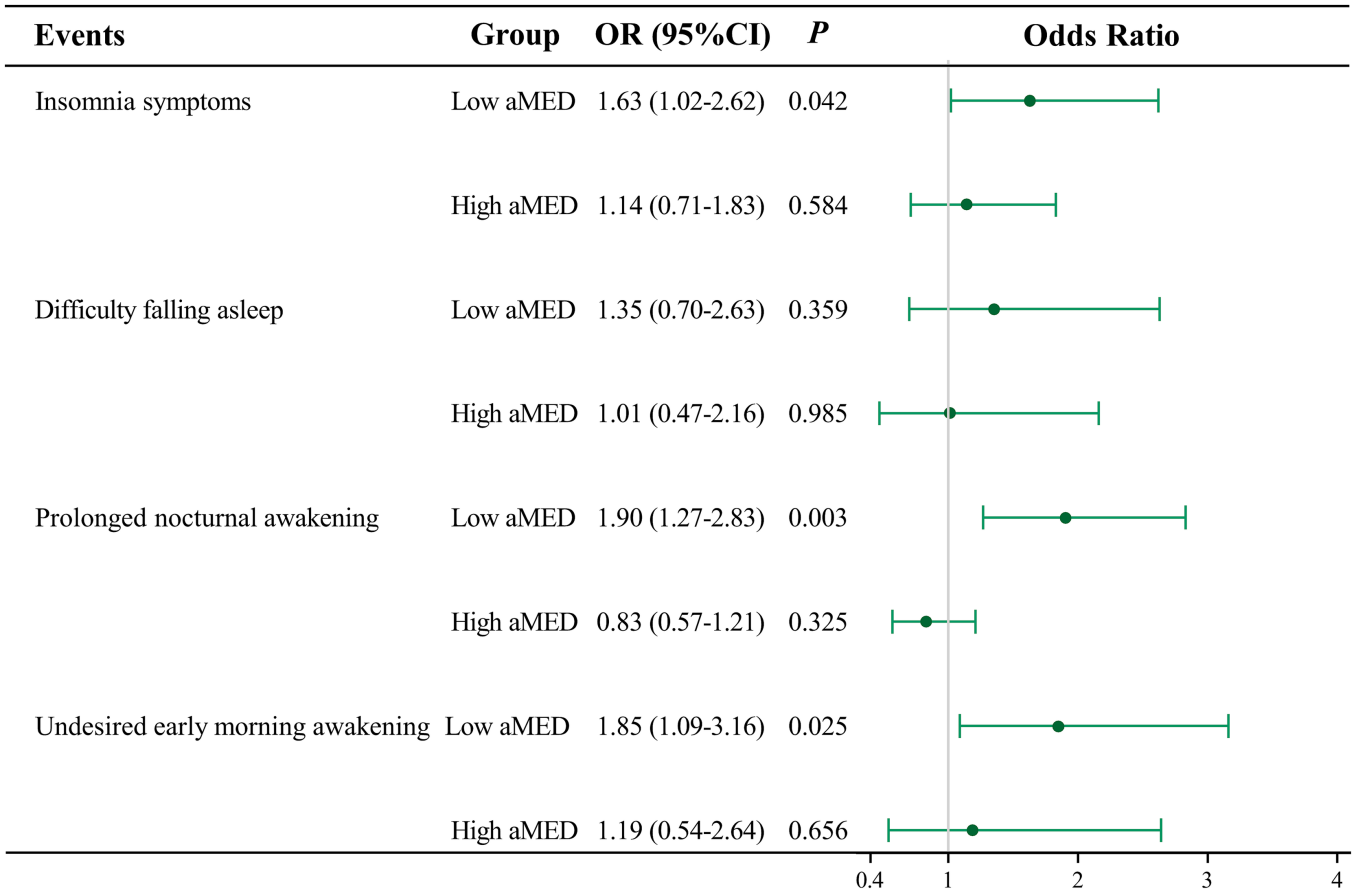
Adherence to the aMED affects the association between sedentary behavior and insomnia symptoms. OR, odds ratio; CI, confidence interval. Insomnia symptoms, adjusted for age, ethnicity, occupation, anxiolytics, sedatives, hypnotics, depression, and smoking status. Difficulty falling asleep, adjusted for age, ethnicity, PIR, occupation, anxiolytics, sedatives, hypnotics, depression, and smoking status. Prolonged nocturnal awakening, adjusted for age, anxiolytics, sedatives, hypnotics, depression, and smoking status. Undesired early morning awakening, adjusted for ethnicity, PIR, occupation, vitamin D, anxiolytics, sedatives, hypnotics, depression, a history of CVD, cancer, smoking status, and diabetes.

Figure 2 shows that adherence to the aMED influenced the association between sedentary behavior and insomnia symptoms. After adjusting for age, ethnicity, occupation, anxiolytics, sedatives, hypnotics, depression, and smoking status, among the postmenopausal women with lower adherence to the aMED, the odds of insomnia symptoms were higher in those with sedentary time of ≥8 h (OR = 1.63, 95% CI: 1.02–2.62). Similarly, among the postmenopausal women with lower adherence to the aMED, sedentary time of ≥8 h was also associated with prolonged nocturnal awakening (OR = 1.90, 95% CI: 1.27–2.83) and undesired early morning awakening (OR = 1.85, 95% CI: 1.09–3.16). Among the women with high adherence to the aMED, no association between sedentary behavior and insomnia symptoms was observed (OR = 1.14, 95% CI: 0.71–1.83). The findings suggest that adherence to the aMED may modulate the relationship between sedentary behavior and insomnia symptoms in postmenopausal women.

## Discussion

Our study investigated the moderating effect of adherence to the Mediterranean diet on the relationship between sedentary behavior and insomnia symptoms in postmenopausal women. Sedentary time of ≥8 h was associated with higher odds of insomnia symptoms, prolonged nocturnal awakening, and undesired early morning awakening. No significant association was observed between adherence to the aMED and insomnia symptoms. In addition, lower adherence to the aMED intensified the association between sedentary behavior and insomnia symptoms in the postmenopausal women.

The relationship between sedentary behavior and insomnia symptoms reported in this study is consistent with previous studies (29, 30). Creasy et al. (29) found that longer durations of sedentary time were associated with shorter sleep duration and poorer sleep quality in postmenopausal women. Similarly, Seib et al. (30) also identified an association between sleep disturbances and sedentary behavior. Modifiable lifestyle factors may indirectly impact sleep by influencing health status. Sedentary behavior can disrupt the body’s internal clock, leading to difficulty falling asleep and maintaining sleep throughout the night (31). Furthermore, sedentary behavior is often associated with increased screen time, particularly in the evening, which can suppress melatonin production and delay sleep onset (32).

In our study, adherence to the aMED dietary pattern was not associated with insomnia in the postmenopausal women. This finding contrasts with previous studies that have reported the beneficial effects of the Mediterranean diet on sleep quality and overall health (13, 33, 34). Gupta et al. (35) indicated that a sleep duration of <7 h per night was associated with a lower aMED score in women. Participants adhering to the Mediterranean diet experienced improved sleep and fewer insomnia symptoms (13, 34). Furthermore, the Mediterranean diet predicted better sleep quality among women living in the U.S. (33). The lack of an association between the Mediterranean diet and insomnia symptoms may be due to various factors, including the complexity of dietary patterns and the multifaceted nature of insomnia. Notably, our finding revealed a moderating effect of adherence to aMED on the association between sedentary behavior and insomnia symptoms in the postmenopausal women. These results highlight the potential significance of a healthy lifestyle and a balanced dietary pattern in improving sleep quality and alleviating insomnia symptoms among postmenopausal women.

The Mediterranean diet is characterized by high consumption of fruits, vegetables, whole grains, and olive oil, all of which possess anti-inflammatory and anti-oxidant properties (19). In contrast, sedentary behavior has been associated with chronic low-grade inflammation, driven by adipose tissue dysfunction and pro-inflammatory cytokine production (36, 37). Therefore, individuals adhering to the Mediterranean diet may experience a reduced inflammatory burden, which could help mitigate the inflammatory response induced by sedentary behavior and ultimately preserve sleep quality. Furthermore, the Mediterranean diet is beneficial for both cardiovascular and metabolic health, which are potential mediators in the relationship between sedentary behavior and insomnia symptoms (38, 39). Therefore, higher adherence to the aMED may help attenuate the physiological processes underlying insomnia symptoms induced by sedentary behavior.

Our findings highlight the importance of promoting both physical activity and dietary interventions to improve sleep quality, particularly in postmenopausal women who may be at an increased risk for insomnia symptoms. Healthcare providers should emphasize the importance of reducing sedentary time and incorporating regular physical activity into daily routines. In addition, encouraging adherence to the Mediterranean diet, which is rich in fruits, vegetables, and whole grains, may offer additional benefits for sleep health in postmenopausal women.

Several limitations should be acknowledged in our study. First, the cross-sectional study design limited the ability to establish causality and temporal relationships between sedentary behavior, aMED adherence, and insomnia symptoms. Longitudinal studies and intervention trials are needed to confirm the observed association and assess the efficacy of higher aMED adherence on sleep quality in postmenopausal women. In addition, information on dietary habits and sedentary behavior was self-reported, which might have introduced recall bias. Future studies employing objective measures, such as accelerometers and dietary records, could provide a more accurate assessment of sedentary behavior and adherence to the dietary pattern. Finally, while self-reported data established the presence of insomnia symptoms, clinical guidelines suggest that the diagnosis of insomnia is primarily based on medical history and that polysomnography is not routinely required for assessment. Future studies are needed to provide a more accurate diagnosis of insomnia.

## Conclusion

Adherence to the aMED may modulate the association between sedentary behavior and insomnia symptoms in postmenopausal women. Sedentary time of >8 h was associated with higher odds of insomnia symptoms, and this association was further exacerbated in the women with lower aMED adherence, highlighting the importance of a dietary pattern in mitigating the adverse effects of sedentary behavior on sleep quality. Future research should explore the potential mechanisms and further validate the effectiveness of lifestyle interventions aimed at reducing sedentary behavior.

## Data Availability

Publicly available datasets were analyzed in this study. This data can be found here: NHANES database, https://wwwn.cdc.gov/nchs/nhanes/.
